# Influence of Haunch Geometry and Additional Steel Reinforcement on Load Carrying Capacity of Reinforced Concrete Box Culvert

**DOI:** 10.3390/ma16041409

**Published:** 2023-02-08

**Authors:** Hafiz Ahmed Waqas, Muhammad Waseem, Abdullah Riaz, Muhammad Ilyas, Muhammad Naveed, Hermann Seitz

**Affiliations:** 1 Department of Civil Engineering, Ghulam Ishaq Khan Institute of Engineering Sciences and Technology, Topi, Swabi 23640, Pakistan; 2 Faculty of Mechanical Engineering and Marine Technology, University of Rostock, Justus-von-Liebig-Weg 6, 18059 Rostock, Germany; 3 Department of Life, Light and Matter, University of Rostock, Albert Einstein-Str. 25, 18059 Rostock, Germany

**Keywords:** critical failure location, culverts, Finite Element Method (FEM), stress concentration

## Abstract

The culverts are used to safely convey water under railways, highways, and overpasses. They are utilized in drainage areas or water channels and in areas where the bearing capacity of soil is low. The design and construction of this crucial infrastructure need to be improved to meet contemporary demands of reliability and affordability. Precast reinforced box culverts are popular alternatives as they ensure strength, durability, rigidity, and economy. This research seeks to develop an effective and affordable design improvement procedure for a precast box culvert using modern numerical tools. The Finite Element Method (FEM) based approach is used in studying the effects of haunch geometry and additional steel reinforcement on the load-bearing capacity of box culverts. A conventional box culvert is analyzed to create the numerical models in the Abaqus FEM code and to investigate the load-bearing capacity of culverts with an expanded span. The outcomes of the study reveal the critical places for stress concentration as well as the location of maximum damage. It is found that haunch geometry and additional reinforcement at these critical places significantly affect the load-carrying capacity of a culvert. From the comparison of capacity curves of models with and without haunches and diagonal reinforcement, it is found that a 25% increase in load-carrying capacity is achievable with the recommended changes. The proposed design improvement technique can be employed for the cost-effective and safe design of a concrete box culvert with larger span lengths and high water-flowing capacities. The findings of this study are expected to assist practitioners in strength enhancement tasks of box culverts for increased structural stability and drainage efficiency.

## 1. Introduction

A culvert is an essential part of a drainage system that helps to manage water flows and provides a dependable course for water to pass through embankments of highways and railways and helps prevent flooding [1]. There are two main types of culverts: rigid and flexible. Rigid culverts, made of concrete, are designed to withstand bending loads with minimal deformation. Flexible culverts, made of steel, are designed to interact with the soil structure and transfer loads differently [2]. The suitability of a culvert type is determined by its ease of construction and required drainage capacity. Box culvert structures are economical since they have immensely high rigidity due to their monolithic action, and these do not require isolated foundations. For small discharges, single-cell or box culverts are used; for large discharges, multi-cell or multi-bin culverts are normally utilized [3]. The top slab of box culverts is designed to support the live loads of a moving vehicle. Similarly, the walls and base slab resist the earth pressure and hydrostatic pressure from outside and from inside, respectively [4].

Climate change and meteorological variations were particularly considered during the original design of old hydraulic structures [5], and whenever there is heavy precipitation, well-planned culverts can drain the water both quickly and effectively. However, outdated or poorly designed culverts cause upstream floods and lead to critical losses and damage [6]. Therefore, the capacity of culverts should be enhanced to ensure adequate water discharging and load-carrying capabilities.

Efforts have been made in the past to develop cost-effective ways of enhancing the drainage and load-carrying capacity of culverts. One approach is the hydraulic modification of existing culverts to increase their capacity rather than completely rebuilding the entire structure. Contemporary design standards assess the performance of a culvert by computing the inflow and outflow of water based on established hydraulic theories while the least efficient flow control is chosen based on anticipated headwater levels. However, by implementing the better design improvement method, the flow and overall performance of the culvert could be improved [7].

In recent years, significant research has been conducted on experimental and numerical analysis and assessment of a box culvert. Crisfeld [8] analyzed the effects of cracking and the failure stress response of a box culvert. Marzouk et al. [9] devised a technique for the determination of the punching and deformation demand of a reinforced concrete (RC) box culvert. Polak [10] employed three-dimensional (3D) shell elements for shear and flexural strength assessment of RC box culverts.

Some researchers experimentally examined the effect of earth pressure on box culverts and compared the results with existing theoretical models to offer design improvements [11,12]. Several academics have studied box culverts to understand the impact of soil-structure interaction on the performance of box culverts [13,14]. Gong et al. carried out experimental and numerical evaluations to study the failure mechanism of reinforced concrete box culverts [15]. Anil and Ali [16] utilized FEM to validate the experimental findings of the box culvert. Moradi et al. [17] validated the experimental works of Maximos et al. [18] and Garg [19] through Finite Element Methods (FEM) and found that FEM tools can simulate the structural behavior of culverts with reasonable accuracy and lesser physical effort. Sinha and Sharma [20] analyzed the design of these small bridges with an emphasis on the specifications for the Indian road congress. Anderson et al. [21] proposed seismic design methods for the culvert. Esra [22] studied the response of long-span corrugated steel box culverts under ultimate loads and proposed a method to adjust the design force calculations. Kim et al. [23] investigated the failure mechanism of box culverts subjected to corrosion by employing FEM. Zenagebriel et al. [24] performed an experimental and numerical investigation on precast RC U-shaped box culverts to examine the effect of various loading conditions both on the slab and sides of the culvert.

Recently, a smart precast box culvert equipped with fiber optic sensors was designed. These sensors were able to detect and measure strain and stress on the culvert in order to monitor structural health via the stress–strain measurement [25]. Additionally, recent work has been focused on reducing the effects of load on box culverts under different loads of construction such as culverts near tunnels or next to train tracks [26,27,28].

Despite these efforts, the studies related to design improvements and load-carrying capacity enhancement of culverts with various strengthening methods have not been explored so far. Against this background, this paper uniquely presents a numerical assessment of a non-conventional box culvert with a larger span length through FEM. The purpose is to study the effects of various strengthening methods and haunch geometry with additional reinforcement on the strength and drainage capacity enhancement of the culvert structure. Furthermore, the FEM is used to simulate the structural behaviors of unstrengthened and strengthened culverts in terms of their deformation response and stress concentrations at critical places of the box culvert using Abaqus software [29].

FEM is an essential technique for predicting events that take place during the fabrication and operation of objects and structures. It has accelerated the advancements in numerous fields of research, such as fluid mechanics, mechanics of structures, fracture mechanics, heat conduction, and biomechanics. It has been proven that this approach, which is labor-intensive to build, calculate, and analyze models, is successful in resolving problems of design improvements [30,31,32]. The Abaqus FEM code has been effectively used in much culvert-related research for design and analysis [16,33,34,35,36,37].

**Figure 1:**
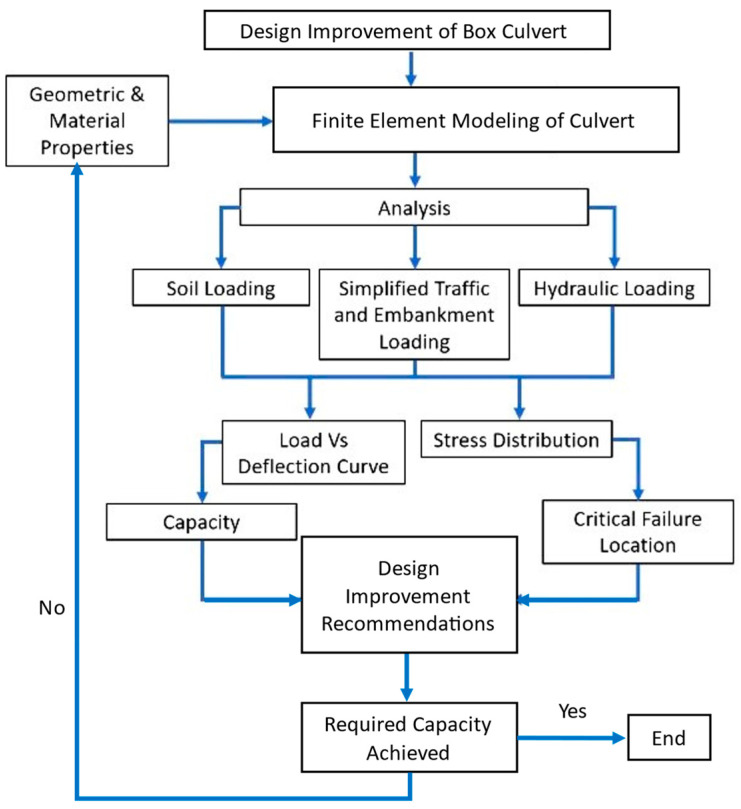
The methodological framework adopted in the current research study.

The inner span of a box culvert is normally about 3.5 m [4]. For larger spans and discharges, the conventional size becomes ineffective and calls for the design of multiple sections thus leading to increased costs and construction efforts. A larger-span culvert is therefore an economical alternative. However, its structural integrity and efficiency need further exploration. This research provides design recommendations for larger-span culverts using the FEM approach. The load-bearing capacity of conventional culverts with an increased span length is evaluated, and the deformed shape, along with the stress distribution of the reference culvert, is utilized to indicate the crucial zones of failure. At these critical failure areas, additional reinforcement and haunch geometry are employed as capacity improvement measures. The efficiency of various strengthening techniques is evaluated on a larger span culvert to obtain the increased design capacity of a culvert for supporting heavier loads and conveying large quantities of water. The adopted analysis mechanism effectively utilizes the FEM techniques to analyze multiple scenarios and conveniently identify an efficient design improvement method for a culvert structure. The presented research work is expected to help practitioners in updating the existing design and analysis techniques and provide an effective methodology for the design enhancement of box culverts to increase both structural stability and drainage efficiency. The complete scheme of this study is depicted in Figure 1.

## 2. Materials and Methods

### 2.1. Model Description

**Figure 2:**
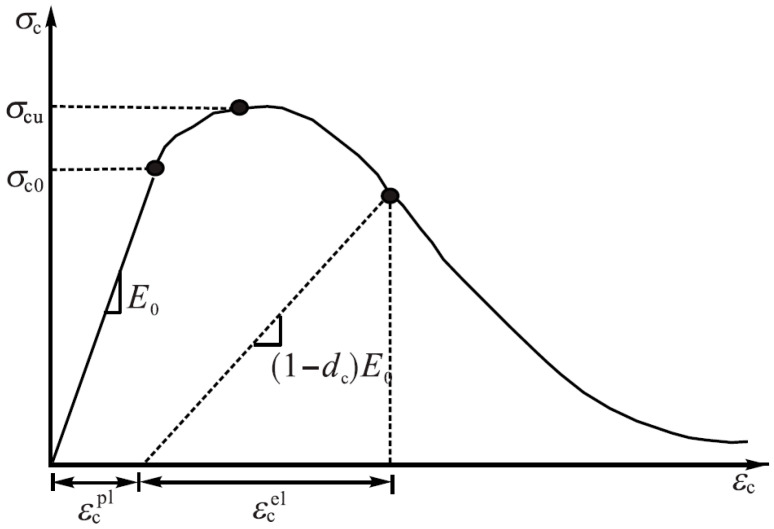
Schematic diagram of the CDP model (image used with the permission of publisher) [39].

The numerical model of the culvert was developed using the geometry and reinforcement details in the Abaqus FEM code for capacity enhancement. Moreover, the Concrete Damage Plasticity (CDP) model was used to simulate the constitutive behavior of concrete [38] as shown in Figure 2.

To simulate the inelastic behavior of concrete, the damaged plasticity model for concrete combines the ideas of isotropic damaged elasticity with isotropic tensile and compressive plasticity. The concrete damage model is a continuous, plasticity-based model. The model is intended for applications in which low confining pressures and monotonic, cyclic, and dynamic stresses are applied to the concrete. This model decomposes the total strain ε into elastic ε^el^ and plastic strains ε^pl^ as follows.

(1) $$\varepsilon ={\;\varepsilon }^{\mathrm{el}}+\varepsilon^{\mathrm{pl}}$$

The following equations give the stress–strain relations under uniaxial compression and tension:

(2) $$\sigma_{t}=\left(1-d_{t}\right)E_{o}\;\left(\varepsilon_{t}-\varepsilon_{t}^{\mathrm{pl}}\right)$$

(3) $$\sigma_{c}=\left(1-d_{c}\right)E_{o}\left(\varepsilon_{c}-\varepsilon_{c}^{\mathrm{pl}}\right)$$

where E_o_ is the initial (undamaged) elastic stiffness of the material, $\varepsilon_{t}$ is total strain, $\varepsilon_{c}^{\mathrm{pl}}$ and $\varepsilon_{t}^{\mathrm{pl}}$ are compression and tensile equivalent plastic strains, and d_t_ and d_c_ are tension and compression damage parameters, respectively.

**Figure 3:**
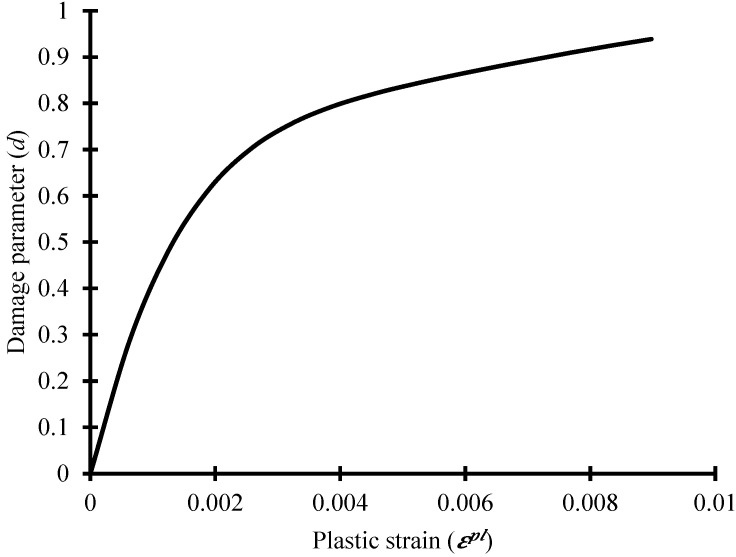
Concrete tension damage vs. cracking strain.

The two primary failure mechanisms inferred by the model are the compressive crushing and tensile cracking of the concrete material. In the plastic-damage model, a plastic-damage variable called “d” was defined. If plastic deformation occurs, the value of this variable increases. The non-dimensional variable “d” has a maximum value of one. When the plastic-damage variable attains its maximum value at a particular location in the solid, the total damage takes place in the form of a macroscopic crack. The two damage parameters for compressive and tensile behavior were used as functions of equivalent plastic strains. The relationship between the tension damage variable and cracking strain is shown in Figure 3. These parameters control the evolution of the yield surface and are related to failure mechanisms under tension and compression loading. The damage parameters can have values between zero and one, where one corresponds to a complete loss of strength and zero to the undamaged material.

The plastic flow of the material was simulated by flow parameters in the concrete damaged plasticity model including dilation angle (ψ), flow potential eccentricity (e), viscosity parameter (μ), and coefficient determining the shape of the deviatoric cross-section (K). The damaged plasticity model assumes non-associated potential flow. The flow potential G follows the Drucker-Prager hyperbolic function:

(4) $$G=\sqrt{{\left({e\sigma }_{t0}\tan\varphi \right)}^{2}+{\bar{q}}^{2}}-\bar{p}\tan\varphi$$

where φ is the dilation angle measured in the p−q plane at high confining pressure, $\sigma_{t0}\;$is the uniaxial tensile stress at failure, and e is referred to as the eccentricity, the rate at which the function approaches the asymptote. These continuous and smooth flow potential parameters ensure that the flow direction is defined uniquely. The function asymptotically approaches the linear Drucker-Prager flow potential at high confining pressure stress and intersects the hydrostatic pressure axis at 90.

The biaxial behavior of concrete material was considered with an initial equi-biaxial compressive yield stress (σ_bo_) and an initial uniaxial compressive yield stress (σ_co_) ratio (σ_bo_/σ_co_). In the case of uniaxial loading, cracks move in a direction opposite to the stress direction and the available load-carrying area decreases as a result of the growth of cracks and rise in the effective stress. The effective load-carrying area is greatly diminished once a significant quantity of material fails. The effective uniaxial cohesion stress at any instance determines the size of the yield surface. The effective cohesion stress in compression and tension is provided below:

(5) $$\bar{\sigma_{t}}=\frac{\sigma_{t}}{\left(1-d_{t}\right)}=E_{o}\left(\varepsilon_{t}-\varepsilon_{t}^{\mathrm{pl}}\right)$$

(6) $$\bar{\sigma_{c}}=\frac{\sigma_{c}}{\left(1-d_{c}\right)}=E_{o}\left(\varepsilon_{c}-\varepsilon_{c}^{\mathrm{pl}}\right)$$

The traffic and embankment loads were modeled as equally distributed loads on the surface, but the soil and water loads were applied as depth-varying triangular loads. To derive load defection curves and stress distributions, the analytical equations were solved using the Abaqus solver of the Dassault Systèmes Simulia Corporation. The load–deflection curve was used to compute the load-carrying capacity, and the stress distribution of the structure was used to pinpoint the critical failure points. The geometry of the haunches and additional steel reinforcement in the optimized culvert structure was utilized to increase its capacity.

### 2.2. Geometric Details

**Figure 4:**
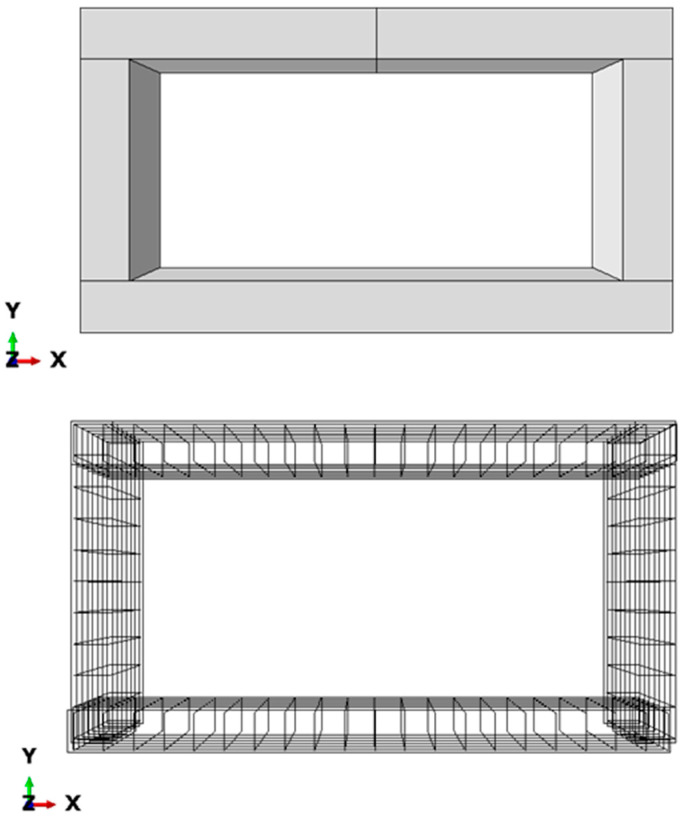
FEM of reinforcement cage and concrete box culvert.

A typical RC box culvert with an extended span of more than 3.5 m was selected as a reference model for this study. The box culvert’s structure is made up of a steel reinforcement cage and a concrete box, as seen in Figure 4. The main and distribution horizontal and vertical bars of the reinforcing cage had rebar sizes of 16 mm. The inner span of the culvert is 4.0 m, and the wall and slab thickness are both preserved at 0.4 m. The normal reinforcing spacing is 200 mm, and the concrete cover thickness is 40 mm.

### 2.3. Materials and Elements

The material properties of concrete for the CDP model utilized are based on past research [39]. These properties are listed in Table 1 for materials such as steel and concrete. Table 2 details the plastic flow parameters of the CDP model.

The elements of concrete and steel are discretized for FEM using 3D wire elements for reinforcement and three-dimensional solid elements for concrete components [24]. The components for reinforcement were 3D, 2 node (T3D2) elements. T3D2 elements are used to simulate slender, line-like structures that only enable axial loading along the element and do not support moments or forces that are perpendicular to the centerline. The concrete elements were meshed with continuum, 3D, 8 node elements with reduced integration (C3D8R). C3D8R elements are linear brick elements with fewer integration points and are standard elements for stress analysis. A courser mesh would yield faster solution convergence while a finer mesh would provide improved results with additional computational cost. The mesh convergence was undertaken by varying the mesh sizes of 200, 100, 50, and 20 mm for concrete, as well as reinforcement, and the results are provided in the section on modal validation.

### 2.4. Boundary Conditions, Interactions, and Loading

The bond between concrete and steel was taken into account using the embedded region method of the Abaqus FEM numerical package. By evaluating the stiffness of the reinforcement elements independently of the concrete elements, this method of modelling the steel reinforcement resolves the mesh restriction issue that arises in the discrete and smeared modelling of reinforcement. With this technique, the host element (concrete) and the slave element are perfectly joined (steel rebar). Additionally, this approach allows for the displacement of the surrounding concrete components to be compatible with the displacement of the steel bars. When applied to complex models, the embedded method is extremely helpful. However, this model expands the number of nodes and degrees of freedom; consequently, it needs more run time and costs more to compute.

**Figure 5:**
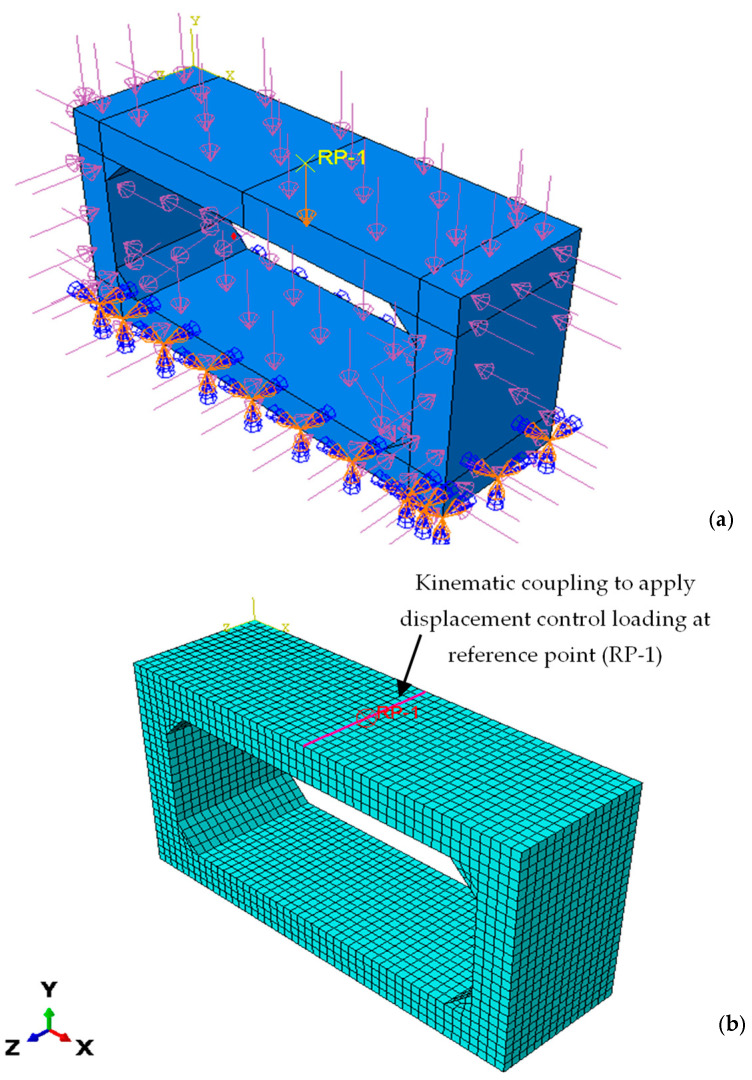
Details of model constrains and loading mechanism; (**a**) model constrains; (**b**) kinematic coupling for displacement-controlled loading.

The culvert’s bottom end was modelled as a fixed foundation using fixed boundary conditions. To simulate field conditions, the remaining surfaces received the necessary weight of soil and water to properly constrain the model with the required boundary conditions. With the appropriate loadings applied to the locations for lateral earth pressure and hydrostatic pressure, the top surface was loaded with a 75 mm layer of asphalt and a 0.3 m layer of soil [40]. The loading conditions and model boundary conditions are shown in Figure 5.

For the performance evaluation of various strengthening methods, the displacement-controlled loading of 120 mm was applied at the reference point to obtain the capacity curve. The motion of one group of nodes on the top slab was restricted by kinematic coupling constraints to rigid body motion defined by a reference node. These restrictions were helpful in controlling the deflection of one node, which was linked to a large number of nodes (the “coupling” nodes). The specification of a reference node, coupling nodes, and the constrained degrees of freedom at these nodes was provided for a kinematic coupling constraint. A kinematic link allowed the reference point (RP-1) and top surface to interact with one another. The load vs. displacement curve was then produced at the reference position by this method.

The critical stress locations in the reference model were sought from the analysis results. These crucial locations were subsequently retrofitted with various strengthening configurations to identify the efficient approach to strength enhancement.

## 3. Results and Discussion

### 3.1. Modal Validation

A numerical model of the culvert was prepared to indicate the effectiveness of the FEM technique and modal validation. The model was constructed based on the experimental work of Maximos et al. [18]. The experimental study used a full-scale precast box culvert for testing as per the American Society of Testing and Materials (ASTM) standards [41,42,43]. The specimen comprised dimensions of 2120 × 1220 × 203 mm where the first two dimensions correspond to inner short- and long-span lengths of culverts, and the third dimension represents the thickness of walls and slabs of a culvert. The tested segment was 1220 mm long, and the reinforcement was provided as per ASTM C1577. The specimen was made with a concrete compressive strength of 52.40 MPa and deformed bars of 470 MPa. The box culvert was subjected to a wheel load at the top of the slab using a footprint plate, and the structure was loaded until failure. The failure occurred when cracks developed in the negative moment sections of the side walls at a load of 244.65 kN (24.5 Tonnes).

**Figure 6:**
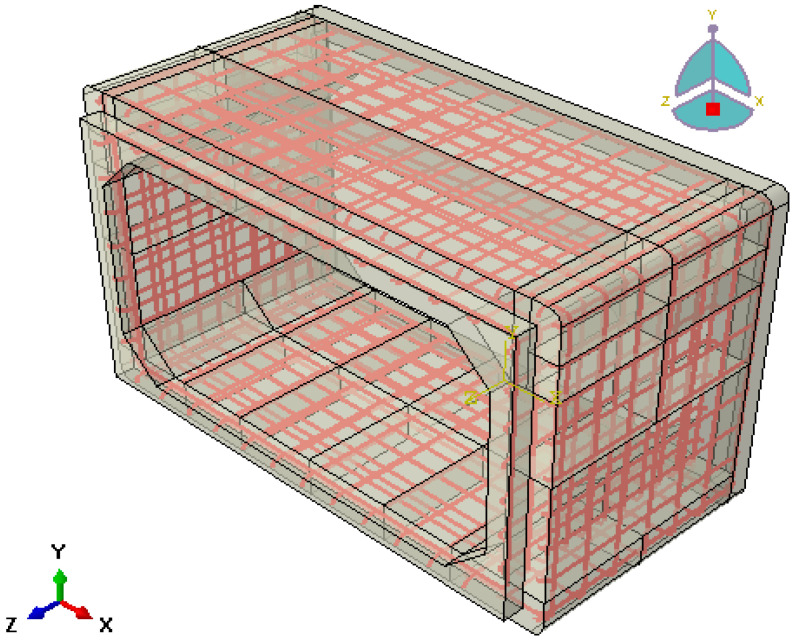
Numerical model based on the experimental work of Maximos et al. [18].

**Figure 7:**
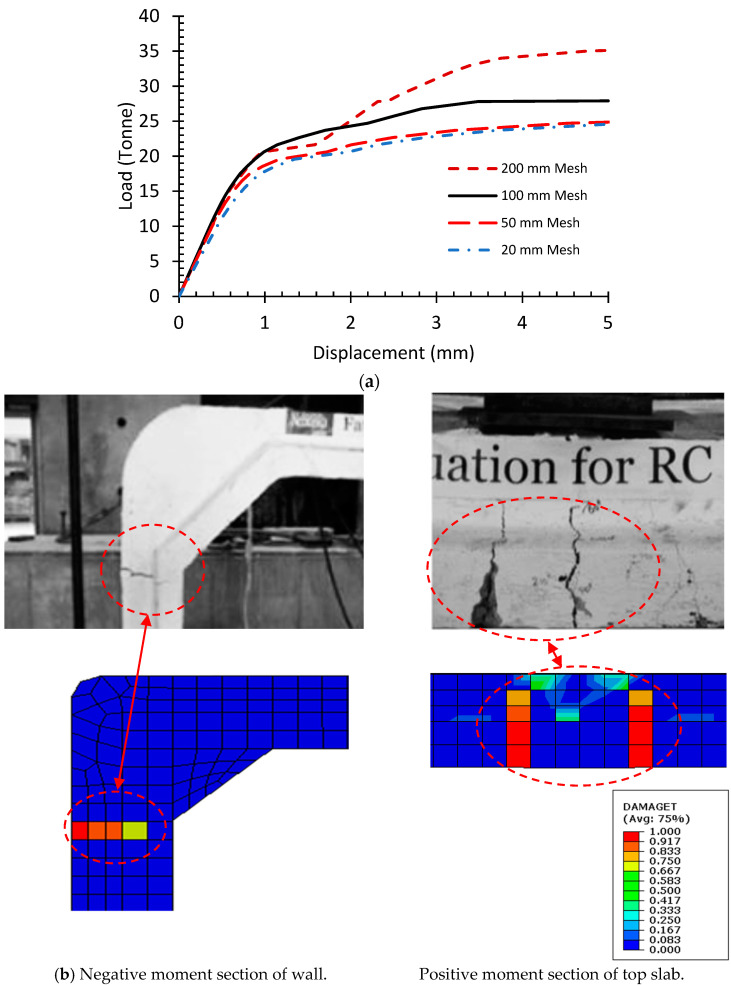
(**a**) Performance of numerical model with different mesh sizes and (**b**) crack patterns observed in the experiment of Maximos et al. [18] (image used with the permission of publisher).

For the numerical modeling of the test specimen, the procedures mentioned in Section 2.1, Section 2.3, and Section 2.4 were utilized to simulate the constitutive behavior of the materials and apply the required modeling parameters. The numerical model of the tested specimen for modal validation is shown in Figure 6. For modal calibration, the load vs. deflection response of the structure with different mesh sizes of steel and concrete was obtained at the point of application of loading. The results of this analysis are provided in Figure 7a, and Table 3 compares the ultimate load and deflection response of structures with different mesh sizes. An analysis of Figure 7a and Table 3 revealed that the error in the results was minimized when the mesh size was set at 50 mm. Moreover, as the mesh size was decreased below 50 mm, the results remained consistent and insignificant variations were observed. The results were not found to be mesh-sensitive due to the small size of the elements; however, the size of the element chosen was slightly larger to reduce the computational time. The corresponding element size of 100 mm was assigned to reinforcing steel and concrete as an optimum arrangement between computational time and modal performance [44]. The mesh size of 100 mm for concrete and steel components provided a comparable ultimate capacity with respect to experimental output. From the experiment, the test specimen exhibited an ultimate capacity of 24.5 tonnes while the FEM reported an ultimate capacity of 27 tonnes at failure. Furthermore, the crack patterns observed in the work of Maximos et al. and the FE model were also found to be similar as presented in Figure 7b. Therefore, the resulting numerical modeling parameters showed good agreement with the experimental results where an experiment/FEM ratio of 0.9 would be obtained and an error value of about 10% could be expected.

### 3.2. Parametric Analysis

The parametric analysis was conducted to evaluate the influence of various parameters on the load-carrying capacity of an RC box culvert. The reference model detailed in Section 2.2 was considered for the parametric study and a total of four parameters were considered in the analysis including the strength of concrete, strength of steel reinforcement, amount of steel reinforcement, and haunch geometry. The culvert size and shape, load applications, and boundary conditions remained unchanged for this parametric analysis. The details of the variables selected for parametric analysis are provided in Table 4.

**Figure 8:**
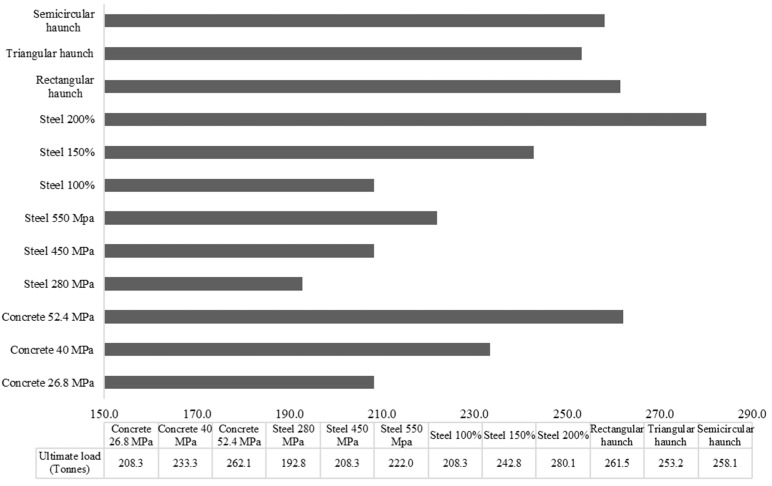
Summary of results of the parametric analysis.

The concrete strength was varied by using concrete mixes of different compressive strengths. Three different concrete strengths of 26.8, 40, and 52.4 MPa were considered. Three different steel strengths of 400, 470, and 550 MPa were included. Similarly, the influence of three different amounts of reinforcement was investigated. Similarly, the amount of reinforcement in the reference model was increased by 150% to 200%. Additionally, the haunch geometry was varied by changing the shape of the haunch. Three different haunch configurations of 300 mm size were studied i.e., a square haunch, a triangular haunch, and a semicircular haunch. The results of the parametric analysis are provided in Table 5 and Figure 8 to identify the influence of each parameter on the ultimate load-carrying capacity of a culvert structure.

A total of 12 analyses were undertaken for four parameters to evaluate their influence on the load-carrying capacity of the RC box culvert. The concrete strength showed a direct impact on the load-carrying capacity of the culvert. The high-strength concrete of double strength could improve the load-carrying capacity by about 25%. Similarly, the steel reinforcement strength and amount also showed a significant impact on the load-carrying capacity of the culvert where the maximum reinforcement amount provided the highest load-carrying capacity, followed by the moderate reinforcement, and the low reinforcement. The 200% increase in the reinforcement amount enhanced the load-carrying capacity by about 35%. Additionally, the haunch geometry showed a significant impact on the load-carrying capacity of the culvert. The provision of haunch geometry could increase the load-carrying capacity by about 20–25%. Overall, the amount of steel reinforcement and the haunch geometry showed a significant impact on the load-carrying capacity of the culvert, followed by the concrete and steel strength.

Since high-strength concrete and steel are more expensive than normal-strength reinforced concrete materials, the combined use of haunch geometry and reinforced concrete material should be encouraged for cost-reduction and effective utilization of resources. This can be realized by identifying the critically distressed areas in the structure where the material performance should be improved.

To identify the efficient approach to strength enhancement, the identified crucial locations were retrofitted with steel reinforcement configurations listed below.

- Case 1: Near Surface Mounted (NSM) L-shaped reinforcement bars;
- Case 2: NSM diagonal brackets;
- Case 3: Concrete haunches and diagonal reinforcement.

**Figure 9:**
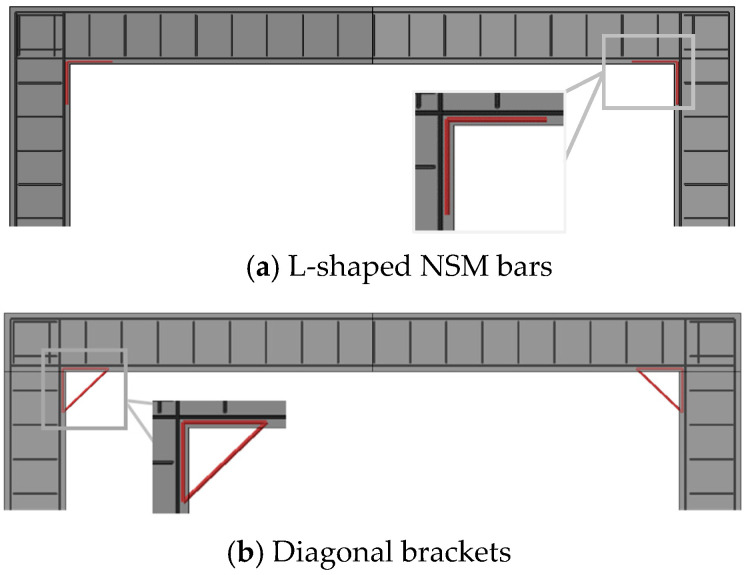
Arrangements of NSM reinforcement.

**Figure 10:**
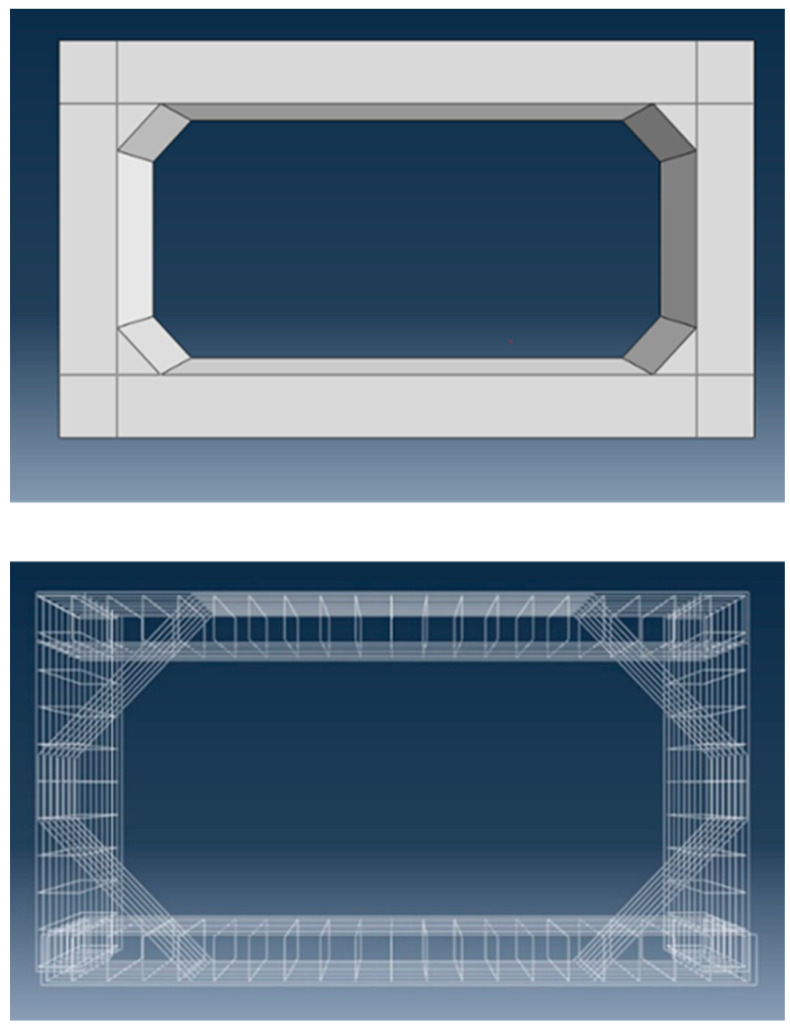
Introduction of concrete haunches and diagonal reinforcement for capacity enhancement.

The rebar size of 16 mm was used for these additional reinforcements. The NSM bars of cases 1 and 2 were embedded in concrete 20 mm from the surface. The spacing of all additional rebars was maintained at 200 mm, similar to the main reinforcement. For case 3, concrete haunches at a size of 0.3 m × 0.3 m were provided at the stress concentration locations. Figure 9a,b show the arrangements of bars for cases 1 and 2, respectively. The graphical representation of concrete haunches and diagonal bars of case 3 are presented in Figure 10. The load–displacement curves of these reinforcement configurations were compared, and their effectiveness was reported to suggest strength-enhancement strategies.

### 3.3. Stress Distribution and Ultimate Load-Carrying Capacity

**Figure 11:**
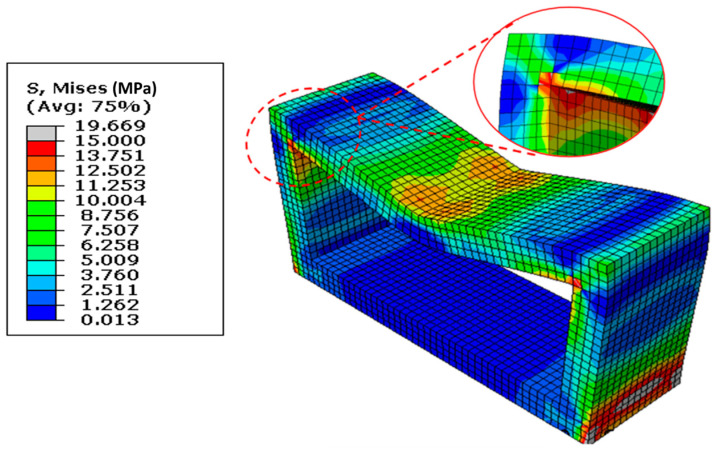
Stress distribution and location of stress concentration.

**Figure 12:**
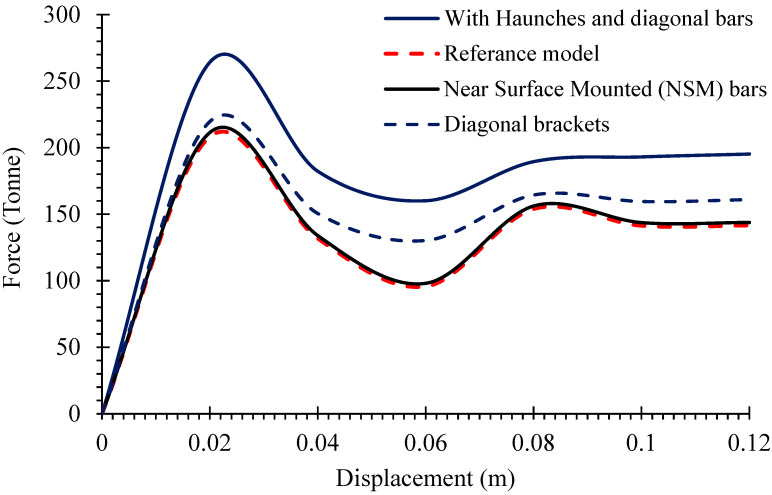
Load vs. displacement curves.

The stress distribution in the reference model under the induced loading is shown in Figure 11. The corner intersection of the culvert’s top slab and walls revealed significant stress concentrations in addition to other typical places. Severe deformation at mid-span and in the joints of the walls and in the top slab was also observed. Excessive mid-span deflection is observed once the load-carrying capacity of the slab is exhausted. The deformation at joint locations further increased the mid-span deflection and reduced the load-carrying capacity. The ultimate load-carrying capacity of the reference culvert was calculated using the load–displacement curve shown in Figure 12. This figure depicts that the culvert structure could bear the maximum load of 210 tonnes with a small deformation of 22 mm. Once the load-resisting capacity of the structure is exhausted due to material damage, the structure experiences extremely large deflections with reduced load-bearing capabilities. The capacity of the reference structure was decreased to 100 tonnes until a 60 mm deformation. The increase in capacity again appeared up to 160 tonnes with a deformation of 80 mm because of the material hardening of steel rebars. No further increase in load-carrying capacity was observed subsequently, and the structure showed continuous large deformations. From Figure 11, it can be observed that the key stressed points are at the intersection of the top slab and walls. Moreover, from the stress distribution and deformed shape, it can be assumed that the strengthening of a culvert structure by means of additional reinforcement and a haunch geometry at the critical position would significantly improve the load-carrying capacity of a longer-span culvert. The strengthened culvert with an extended span would provide better water-flowing capability and would be able to withstand more loads.

### 3.4. Influence of Additional Steel Reinforcement and Concrete Haunches

In order to carefully follow the advancements in structural behavior and overall capacity, the influence of additional steel reinforcement and concrete haunches was investigated by comparing the load-displacement curves of the reference model and strengthened structures. The procedure described in the preceding section was utilized to determine the capacity of the retrofitted structure. The improved capacity curves of different cases are shown in Figure 12, next to the reference capacity curve. The results indicate that the installation of L-shaped NSM reinforcement would not be beneficial to improve the load-carrying capacity when compared to a diagonal bracket arrangement, which exhibited slight improvement in the capacity of the reference structure. The concrete haunch structure and additional diagonal reinforcements were found to be the most effective, providing a capacity of 270 tonnes. The culvert structure, strengthened with concrete haunches and diagonal bars, has the ability to increase the box culvert’s load-bearing capacity by around 25%. The difference between the peak load-carrying capacity of reference (208.3 tonnes) and the strengthened culvert (270 tonnes) was used to obtain this amount.

To summarize, this study evaluated the impact of different reinforcing methods on the load-carrying capacity of a culvert with an increased span length, which has not been undertaken to date. The NSM strengthening method has been adopted in past studies for various structural components such as beams and has shown some beneficial effects in enhancing structural performance [44]. However, in the case of culverts, this is a unique study and the first of its kind. The performance of NSM reinforcement in the capacity enhancement of a culvert was found to be insignificant compared to concrete haunch geometry and diagonal reinforcement. The current study uniquely identifies the performance of concrete haunch geometry and the diagonal reinforcement-based strengthening method and highlights the significance of the proposed design improvement method.

**Figure 13:**
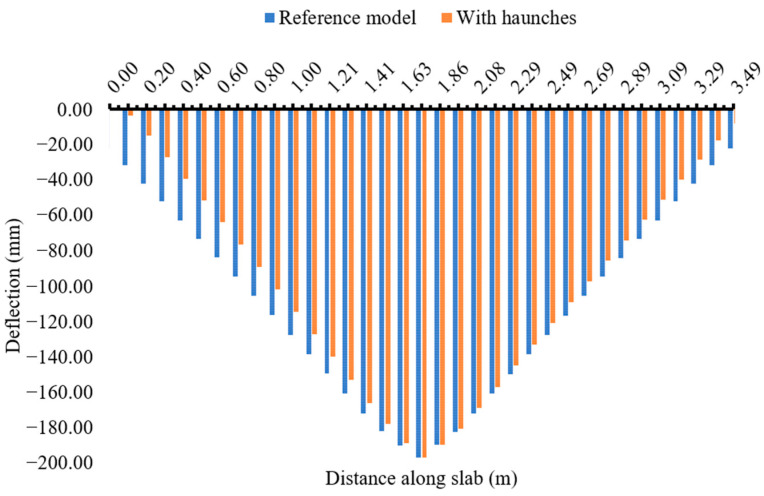
Comparison of deflections in the slabs of reference and haunched culverts.

**Figure 14:**
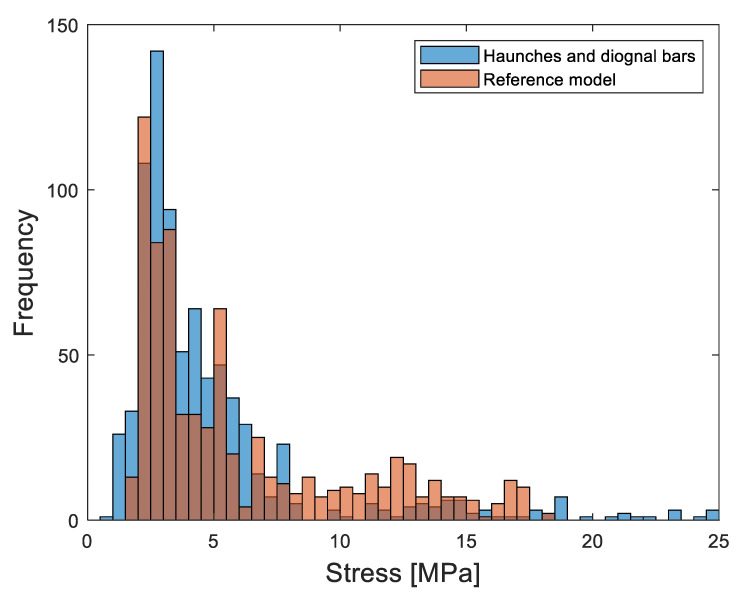
Stress distribution in the wall and slab joints of reference and haunched culverts.

The haunch geometry and additional diagonal reinforcement played a crucial role in enhancing the structural resistance at the location of stress concentration and preventing the box culvert from excessive deformations. Figure 13 provides the comparison of induced deflections of 200 mm in the slabs of reference and haunched culverts. At the mid-span location, both reference and stiffened structures were forced with the same amount of deflection, and hence the deflections were almost similar; however, the deflection is reduced towards the wall and slab joint due to the provision of the haunch and diagonal reinforcement. The haunch and diagonal reinforcement at the joints provided additional stiffness at the joint location where the deflection in the stiffened structure is almost zero. The concrete haunch and diagonal bars also played a vital role in reducing the stress concentration at the critical joint location as shown in Figure 14. The quantity of elements experiencing a high number of stresses in reference culverts is reduced by adopting retrofitting measures. The quantity of elements with a smaller number of stresses is also increased in a retrofitted culvert which indicates that the stresses are well distributed in different elements of joints with lower absolute maximum values. The proposed methodological framework of the strength enhancement of culverts systematically identified the weaker zones in the structure and recommended the most effective strength enhancement method for culverts. The proposed framework presented in Figure 1 could be repeated to achieve the desired load-carrying capacity of a culvert structure with extended span lengths.

## 4. Conclusions

This study evaluated the impact of different strengthening methods on improving the load-carrying capacity of a culvert with increased span length and larger water-flowing capacity. The presented work demonstrated the effectiveness of the FEM for incorporating the required design improvements based on the output of the analysis. A typical culvert structure with an enlarged span was numerically modeled to act as a reference structure. NSM steel rebars and concrete haunches with diagonal strengthening methods were employed at critical failure locations of the structure for design improvement and strength enhancement of the reference model. The selected strength enhancement approaches had been commonly used in previous studies for various structural components, and the present work uniquely explored the application of these methods on culvert structures. The models with NSM reinforcement were found to be insignificant in the capacity enhancement of culverts compared to models where concrete haunch geometry and diagonal reinforcement were considered. The load–deflection curves of the reference model and the model strengthened with different strengthening methods were compared to obtain the ultimate load-carrying capacities of different models. The deformed shapes of the structure and stress distribution of a reference culvert and the culvert strengthened with the most effective design improvement methods were also considered to highlight the performance of a recommended approach of capacity enhancement. The following conclusions were drawn from the current study:

- The link between the slab and the wall was found to have the highest likelihood of stress concentrations;
- The NSM rebars of an L-shape and diagonal bracket slightly influence the capacity and could be useful for small repairs and restoration works;
- The precast reinforced box culvert’s FE modelling indicates that adding diagonal reinforcement and a haunch construction could appreciably enhance its capacity. A 25% increase in the ultimate load-carrying capacity was found when the ultimate capacity of models with and without haunches was compared.

To fully understand the relevance of the suggested design improvement technique for cost-effective and safe design, the performance of box culverts with wider spans and under seismic loadings must also be examined. Furthermore, the influence of other retrofitting configurations, such as the Fiber Reinforced Polymer (FRP), to investigate strength enhancements should also be explored.

## Figures and Tables

**Table 1:** Material information of the study culvert [39].

Details	Concrete	Steel
Density (Kg/m^3^)	2400	7850
Compressive strength (MPa)	26.8	450
Tensile strength (MPa)	2.39	450
Poisson’s Ratio	0.2	0.3
Modulus of elasticity (MPa)	32,500	200,000
Post yielding modulus of elasticity (MPa)	-	20,000

**Table 2:** Plasticity flow parameters for CDP model [39].

Parameters	Values
Dilation angle (ψ)	40°
Eccentricity (e)	0.1
Ratio of biaxial to uniaxial compressive yield stress (σ_bo_/σ_co_)	1.16
Coefficient determining the shape of the deviatoric cross-section (K)	0.66
Viscosity parameter (μ)	0

**Table 3:** Results of model calibration.

Mesh Size		Displacement	Force
Concrete	Steel	mm	Tonnes
200	200	5	35
100	100	3.45	27
50	50	4.5	24.0
20	20	4.5	23.9
Selected mesh size	100 mm for concrete and steel	% Error in results of numerical analysis	10

**Table 4:** Variables for parametric analysis.

Parameter	Variables	Units
Strength of concrete	26.8, 40, 52.4	MPa
Strength of steel	280, 450, 550	MPa
Amount of steel	100, 150, 200	Percent
Haunch geometry	square, triangular, and semicircular	-

**Table 5:** Results of parametric analysis.

Parameter	Variable			Units
Strength of concrete	26.8	40	52.4	MPa
Ultimate load	208.3	233.3	262.1	Tonnes
Increase in capacity	0	12	25.8	Percent
Strength of steel	280	450	550	MPa
Ultimate load	192.8	208.3	222	Tonnes
Increase in capacity	−7.4	0	6.6	Percent
Amount of steel	100	150	200	Percent
Ultimate load	208.3	242.8	280.1	Tonnes
Increase in capacity	0	16.6	34.5	Percent
Haunch geometry	square	triangular	semicircular	-
Ultimate load	261.5	253.2	258.1	Tonnes
Increase in capacity	25.6	21.6	23.9	Percent

## Data Availability

Not applicable.
